# *LSSR1* facilitates seed setting rate by promoting fertilization in rice

**DOI:** 10.1186/s12284-019-0280-3

**Published:** 2019-05-09

**Authors:** Xiaojiao Xiang, Peipei Zhang, Ping Yu, Yingxin Zhang, Zhengfu Yang, Lianping Sun, Weixun Wu, Riaz Muhammad Khan, Adil Abbas, Shihua Cheng, Liyong Cao

**Affiliations:** 1 Key Laboratory for Zhejiang Super Rice Research and State Key Laboratory of Rice Biology, China National Rice Research Institute, Hangzhou, 310006 China; 2 National Key Laboratory of Crop Genetic Improvement, Huazhong Agricultural University, Wuhan, 430070 China

**Keywords:** Cellulase, Microsporogenesis, Secretory protein, CRISPR-Cas9 system, Spikelet sterility

## Abstract

Seed setting rate is one of the major components that determine rice (*Oryza sativa* L.) yield. Successful fertilization is necessary for normal seed setting. However, little is known about the molecular mechanisms governing this process. In this study, we report a novel rice gene, *LOW SEED SETTING RATE1* (*LSSR1*), which regulates the seed setting rate by facilitating rice fertilization. *LSSR1* encodes a putative GH5 cellulase, which is highly conserved in plants. *LSSR1* is predominantly expressed in anthers during the microsporogenesis stage, and its encoded protein contains a signal peptide at the N-terminal, which may be a secretory protein that stores in pollen grains and functions during rice fertilization. To explore the physiological function of LSSR1 in rice, loss-of-function mutants of *LSSR1* were created through the CRISPR-Cas9 system, which showed a significant decrease in rice seed setting rate. However, the morphology of the vegetative and reproductive organs appears normal in *lssr1* mutant lines. In addition, *lssr1* pollen grains could be normally stained by I_2_-KI solution. Cytological results demonstrate that the blockage of fertilization mostly accounted for the low seed setting rate in *lssr1* mutant lines, which was most likely caused by abnormal pollen grain germination, failed pollen tube penetration, and retarded pollen tube elongation. Together, our results suggest that *LSSR1* plays an important role in rice fertilization, which in turn is vital for maintaining rice seed setting rate.

**Electronic supplementary material:**

The online version of this article (10.1186/s12284-019-0280-3) contains supplementary material, which is available to authorized users.

## Background

Rice (*Oryza sativa* L.), the staple food of more than half of the world’s population particularly distributed in Africa and Asia, is critical for global food security (Seck et al. 2012). Panicle number, grain number per panicle, grain weight, and seed setting rate are the major components that determine rice yield. In the past few decades, excellent progress has been made in illustrating the molecular mechanisms of panicle number (Li et al. 2003; Takeda et al. 2003; Xu et al. 2012; Wang et al. 2017), grain number per panicle (Ashikari et al. 2005; Wu et al. 2016; Huo et al. 2017), and grain weight (Li et al. 2011; Ishimaru et al. 2013; Duan et al. 2017). Although recent results have shed some light on the molecular regulation of the seed setting rate in rice (Zhou et al. 2011; Li et al. 2013; Zhu et al. 2017), much remains unknown. Decreased seed setting rate has become a bottleneck that limits further improvement in rice grain yield using hybrid rice (Dan et al. 2014; Li et al. 2016). Additionally, climate variation may result in huge losses of rice yield, since the seed setting rate is susceptible to environmental conditions. Therefore, identifying more genes that control rice seed setting rate and detailing their genetic mechanisms, as well as their application in rice product is of critical importance for the maintenance and improvement of rice yield.

Many factors can lead to spikelet sterility. These include defected pollen grains, abnormal embryo sacs, inappropriate temperature at the reproductive stage, etc. All of these could in turn result in a low rice seed-setting-rate. Successful double fertilization is the first guarantee for spikelet fertility. The process of fertilization in angiosperms goes as follows. Pollen grains land on the stigma, hydrate, and germinate to form a specialized structure called a pollen tube through cell-cell interaction with the pistil (Yadegari and Drews 2004; Palanivelu and Johnson 2010). Pollen tubes then penetrate the stigma and style and reach the transmitting tract (TT) (Jiang 2005). Next, pollen tubes elongate through the TT under the guidance of the pistil to reach the ovules (Márton and Dresselhaus 2010). Finally, the pollen tubes traverse the micropyle and synergid cells, rupture, and release the two sperms to finish double fertilization (Sandaklie-Nikolova et al. 2007; Leydon et al. 2015; Ge et al. 2017). *PSS1* is a kinesin-1 family gene, which plays an important role in regulating the seed setting rate of rice by controlling male meiotic chromosomal dynamics, male gametogenesis, and anther dehiscence (Zhou et al. 2011). Rice *PTB1*, encoding a RING-type E3 ubiquitin ligase, positively regulates the seed setting rate by facilitating pollen tube growth and is affected by the environmental temperature (Li et al. 2013). OsCNGC13, a cyclic nucleotide-gated channel protein, acts as a novel maternal sporophytic factor required for stylar [Ca^2+^]_cyt_ (cytoplasmic calcium concentration) accumulation, ECM (extracellular matrix) component modification, and STT (style transmission tissue) cell death, thus promoting the pollen tube elongation and seed setting in rice (Zhu et al. 2017). Nevertheless, little is known about the correlation between rice seed setting rate and other steps.

During the step in which the pollen tubes penetrate the stigma, they must first penetrate the cuticle and cell wall to enter the papillar cell (Jiang 2005). Wall hydrolases and wall-modulating proteins are known either to be present, derived from the tapetum cells, or to be temporarily secreted in the pollen grains (Suen et al. 2003). They could be specifically synthesized for hydrolysis of the stigma wall, or the tube track wall for pollen tube growth (Suen and Huang 2006). Alternatively, they might control the structure and strength of the expanding pollen tube tips (Bosch and Hepler 2005). The rice cell wall is a type II cell wall, which consists of cellulose, arabinoxylans, glucuronoarabinoxylan, and so on (Carpita and Gibeaut 1993). Glycosyl hydrolases (GHs) in rice pollens during pollen tube growth may act on these polysaccharides in the stigma wall, facilitating the entry of the pollen tubes into the pistil. GHs can be grouped into “clans” according to their three-dimensional (3D) structures (Henrissat and Davies 1997). Clan A is the largest group, which contains a core (β/α)_8_ architecture with an acid/base and a nucleophile on the ends of β-strands 4 and 7 (Jenkins et al. 1995). Glycosyl hydrolase family 5 (GH5) is one of the clan A families that contains enzymes with a wide range of catalytic activities, including cellulases, endo-β-1, 4-xylanase, β-mannosidase, chitosanase, β-primeverosidase, etc... In maize (*Zea mays L.*), the GH5 xylanase *Zm*XYN1 has been proved to facilitate the penetration of pollen tube into silk during sexual reproduction (Suen and Huang 2006). GH5BG is a GH5 β-glucosidase containing a fascin-like domain, which can be up-regulated in response to stress in rice seedlings (Opassiri et al. 2007). However, no study on the physiological role of rice GH5 cellulases has ever been reported.

Here we report a new rice gene, *LOW SEED SETTING RATE1 (LSSR1)*, which is predicted to encode a GH5 cellulase and regulates the seed setting rate of rice. *LSSR1* is predominantly expressed in the rice anther from the premiotic stage to the single-cell pollen stage. Loss-of-function mutation of *LSSR1* achieved through the CRISPR-Cas9 system has no effect on the morphology of the vegetative and reproductive organs, as well as the pollen I_2_-KI staining in rice. Nonetheless, the mutation in *LSSR1* resulted in either a decreased number or none of the pollen tubes reaching to the ovule during fertilization, leading ultimately to a low seed setting rate. The results suggest that *LSSR1* plays a critical role in the process of rice fertilization.

## Materials and methods

### Materials and growth conditions

All materials in this study were with a Nipponbare (*Oryza sativa Japonica*) background. Nipponbare (WT) and *lssr1* mutant lines were grown in experimental fields at the China National Rice Research Institute in Hainan or Hangzhou during the normal growing seasons.

### RNA isolation, cDNA synthesis, and qRT-PCR

Total RNA was extracted from various rice tissues using the RNAprep Pure Plant Kit (Tiangen, China) according to the manufacturer’s protocol. For qRT-PCR analysis, isolated total RNA was first adjusted to the same concentration and then reverse-transcribed with the ReverTra Ace® qPCR RT Master Mix with gDNA Remover (Toyobo, Japan). qRT-PCR was performed with a LightCycler 480 (Roche, Germany) using LightCycler® 480 SYBR® Green I Master Mix (Roche, USA) in accordance with the manufacturer’s instructions. Measurements were obtained using the relative quantification method (Livak and Schmittgen 2001). The rice gene *OsActin* (*LOC_Os03g50885*; http://rice.plantbiology.msu.edu/) was used as the internal control (Zhang et al. 2017). Each reaction was performed with three replicates. Primers used for qRT-PCR are listed in Additional file 1: Table S1.

### Phylogenetic analysis

The protein sequence of *LSSR1* (*LOC_Os02g38260*) was used as a BLAST query to identify putative homologs on the NCBI database. Protein sequence alignments were made using ClustalW with default parameters and analyzed by GENEDOC. The alignment was used to construct a maximum likelihood phylogenetic tree with 500 bootstrap replications, a Poisson model, and other default parameters. The signal sequence was predicted with the TargetP 1.1 Server (http://www.cbs.dtu.dk/services/TargetP/). The structure, molecular model, and active sites of LSSR1 were predicted using Phyre2 (http://www.sbg.bio.ic.ac.uk/phyre2/html/page.cgi?id=index) by comparing with *Pyrococcus horikoshii* endocellulase c2zunB (Wass et al. 2010; Kim and Ishikawa 2011; Kelley et al. 2015). The three-dimensional structure was analyzed by software VMD.

### Subcellular localization of LSSR1 through transient expression in *N. benthamiana*

The full-length CDS of *LSSR1* was amplified from the wild-type cDNA and fused with the 5′ terminus and 3′ terminus of the coding sequence of *EGFP* (*Bam*HI-*Hind*III sites) in pYBA 1132 and pYBA 1152 vectors, respectively (Liu et al. 2017). The recombinant proteins were transiently expressed or co-expressed with mCherry alone in *N. benthamiana* leaves. Confocal imaging analysis was performed using a laser scanning confocal microscope (ZEISS LSM 750, Germany) 3–5 days after infiltration. Primers used for the generation of these fusion constructs are listed in Additional file 1: Table S1.

### Construction of *lssr1*

The *lssr1* mutants were generated using the CRISPR/Cas9 system according to the methods of previous studies (Miao et al. 2013; Huang et al. 2017). Three CRISPR targets for *LSSR1* were selected using CRISPR-P v2.0 (http://cbi.hzau.edu.cn/CRISPR2/). The spacers were cloned by ligating complementary oligos into a type II restriction site (*Bsa*I). Gateway recombination was used to incorporate guide sgRNA into vector pBWA(V)H_cas9i2, which contains *Cas9* driven by *P35S* for expression. The recombinant vectors were then transferred into the Nipponbare callus for the culture of transgenic lines. The genomic DNA of transgenic lines was extracted using the modified CTAB protocol (Murray and Thompson 1980). The genomic region surrounding the CRISPR target site for *LSSR1* was amplified and sequenced to screen for mutants. Mutation identification was done using SeqMan or DSDecode (http://dsdecode.scgene.com/). For mutants with a mutation in the first CRISPR target, homozygous T_3_ plants without T-DNA were used for phenotyping. The transgene-free mutant individuals were identified using the CRISPR/Cas9 vector specific primer. All primers used for sequencing and T-DNA detection are listed in Additional file 1: Table S1.

### Characterization of the mutant phenotype

The plant morphology of the WT and *lssr1* mutant lines was photographed using a digital camera (Nikon HB-40, Japan). Florets and mature panicles were scanned on a scanner (Epson Perfection v330 Photo, Indonesia). Pistils, stamens, and I_2_-KI stained pollen grains were imaged using a microscope (LEICA DM4 B, Germany). I_2_-KI staining was used to primarily evaluate pollen viability with I_2_-KI staining buffer that contained 1% (*w*/*v*) I_2_ and 8% (w/v) KI. FAA (containing an 18:1:1 (by vol.) mixture of 70% ethanol, formalin and acetic acid)-fixed and 70% ethanol-stored panicles at different developmental stages were used for the morphologic image of 3–4, 4–5, and 5–6 mm florets. To examine the seed setting rate of the homozygous T_3_ lines L1–1~L1–5, at least 6 morphology-similar individuals per line with 5 main panicles per plant were used. For the T_0_ individuals with mutation in the 2 and 3 target sites, the transgenic individual without mutation in the target sites was used as the control, as its growth period showed the same with those mutants.

For scanning electron microscopy (SEM) observation, mature anthers of the WT and *lssr1* mutants were pre-fixed in 0.1 M sodium phosphate buffer containing 2.5% glutaraldehyde (pH = 7.0) overnight at 4 °C, then rinsed thrice using 0.1 M phosphate buffer (pH = 7.0). The rinsed samples were post-fixed for 1.5 h in 2% OsO_4_ (in PBS, pH = 7.2) and rinsed thrice again, following gradient ethanol dehydration. The dehydrated samples were processed for critical point drying using a critical point dryer (Hitachi Model HCP-2, Japan), and gold coated by an ion sputter (Hitachi Model E-1010, Japan). The gold-coated samples were observed using a scanning electron microscope (Hitachi Model SU-8010, Japan).

Co-segregation analysis between the phenotype with the genotype in the segregated T_2_ population of L1–2 and L1–3 was conducted to confirm that it was the mutation of *LSSR1* that resulted in low seed setting rates in *lssr1* mutant lines. For genotyping, a CAPS (Cleaved polymorphic amplified sequence) primer 38260 s1-F/R was used for amplification and the restriction enzyme *Bse*NI was used for digestion (Additional file 1: Table S1). The digested products were separated on a 1% agarose gel. To evaluate that the low seed setting rate in *lssr1* lines resulted from their impaired male or female gametophytes, reciprocal crosses between the WT and *lssr1* lines were conducted by artificial emasculation and hand pollination for three consecutive days.

### Aniline blue staining of pollinated pistils

Aniline blue staining was conducted based on previous research with some modification (Zhang et al. 2018). More than 30 pollinated pistils of the WT and *lssr1* lines were collected and fixed in the FAA fixing reagent. The fixed samples were then rinsed with distilled water and incubated in 10 mol·L^− 1^ NaOH for 7 min at 56 °C. Then the softened samples were rinsed with distilled water and stained in a 0.1% (*w*/*v*) aniline blue solution for 12 h. Finally, the samples were examined using a fluorescence microscope (LEICA DM4 B, Germany) with DAPI channel. During the flowering time, the floret was designated to be flowering as long as its glumes open, and the flowered floret was marked for the following experiment.

## Results

### *LSSR1* is predominantly expressed in anthers during the stage of microsporogenesis

A previous microarray analysis suggested that *LOW SEED SETTING RATE 1* (*LSSR1*; *LOC_Os02g38260*) is predominantly expressed in meiotic and single-cell pollen stage anthers (Deveshwar et al. 2011), while the data on the Rice eFP Browser (http://www.bar.utoronto.ca/efprice/cgi-bin/efpWeb.cgi) shows that the expression of *LSSR1 *in meiotic panicles is not dominant (Additional file 1: Figure S1).

**Fig. 1:**
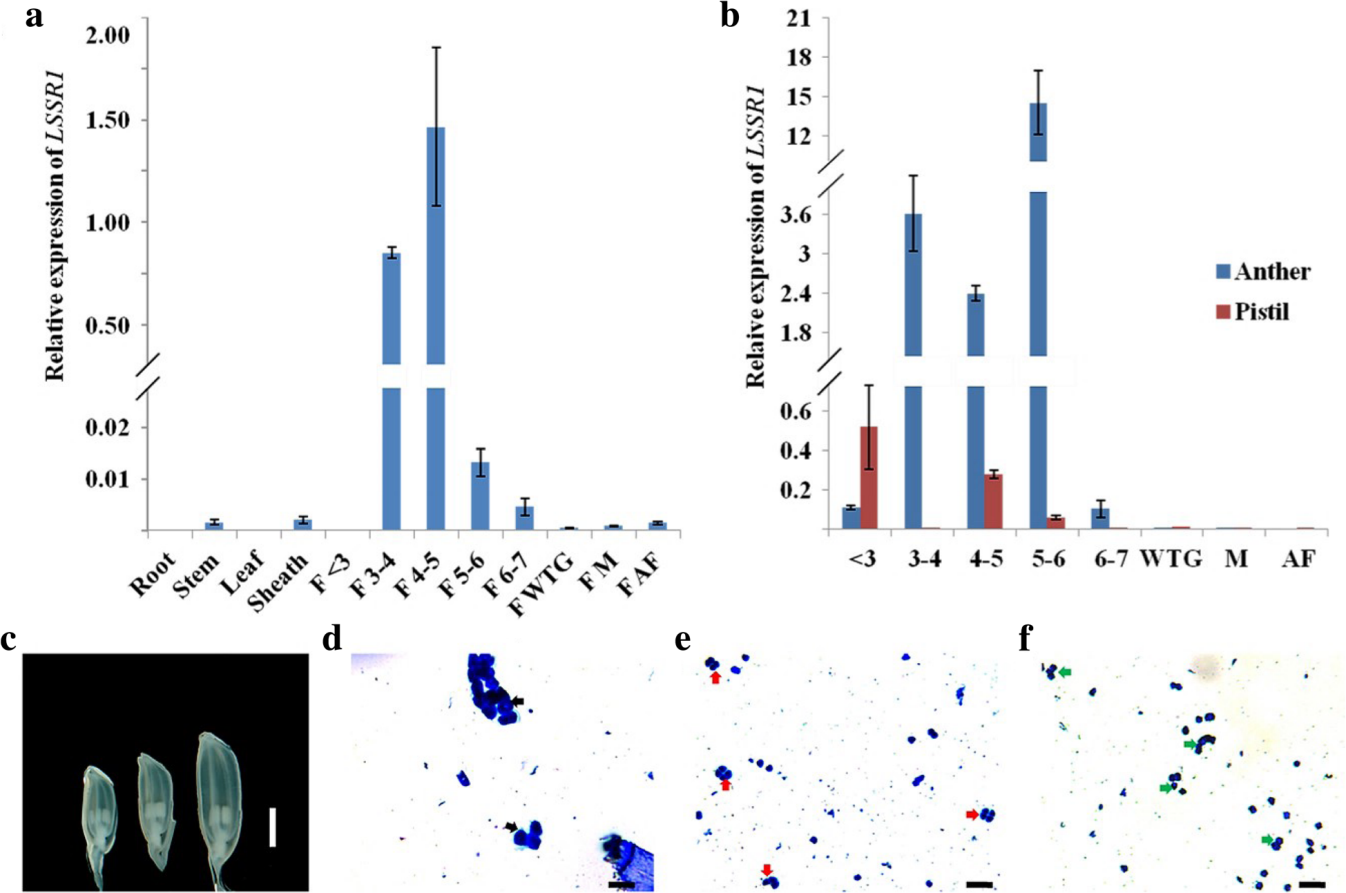
Expression profile of *LSSR1.* **a** qRT-PCR analysis of *LSSR1* in different organs of Nipponbare. F means florets; < 3, 3–4, 4–5 5–6, and 6–7 depict the floret length (mm); WTG indicates that the floret color turns to green from white; M means florets with mature reproductive organs; AF represents florets at 5–10 min after flowering. **b** qRT-PCR analysis of *LSSR1* in anthers and pistils dissected from the florets at different developmental stages. Data are means ± 3SD from 3 replicates. **c** Morphology of 3–4, 4–5, and 5–6 mm florets. **d-f** Aniline blue staining of the microsporocytes (**d**, black arrow), tetrads (**e**, red arrow), and single-cell pollen grains (**f**, green arrow) in 3–4, 4–5, and 5–6 mm florets, respectively. Bars: c, 2 mm; d-f, 50 μm

To confirm the expression pattern of *LSSR1*, qRT-PCR was conducted using total RNA extracted from different rice tissues. Traces of *LSSR1* transcripts were detected in rice stems, sheaths, florets turning to green (FWTG), florets with mature reproductive organs (FM), and florets at 5–10 min after flowering (FAF) (Fig. 1a). Slightly more *LSSR1* transcripts were detected in 5–6 mm florets and 6–7 mm florets (Fig. 1a). Dominant expression of *LSSR1* was detected in 3–4 mm florets and 4–5 mm florets, which were at the premeiotic and meiotic stages, respectively (Fig. 1a, c-e). To more precisely determine these spatial and temporal patterns, *LSSR1* transcripts were examined in separated anthers or pistils from the florets at different developmental stages. The most prominent expression of *LSSR1* was detected in anthers from the 5–6 mm florets, which were at the single-celled pollen stage (Fig. 1 b, c, f). Secondary expression of *LSSR1* was detected in anthers from the 3–4 mm florets and 4–5 mm florets, which were at the premeiotic and meiotic stages, respectively (Fig. 1b, c-e). Although slightly high expressions of *LSSR1* were found in pistils from < 3 mm florets and 4–5 mm florets, respectively, they were greatly lower than that in the microsporogenesis-stage anthers (Fig. 1b). These results indicate that *LSSR1* is mainly expressed in the anthers during microsporogenesis, including the premeiosis, meiosis, and single-cell pollen stages.

### Gene cloning and sequence analysis of LSSR1

**Fig. 2:**
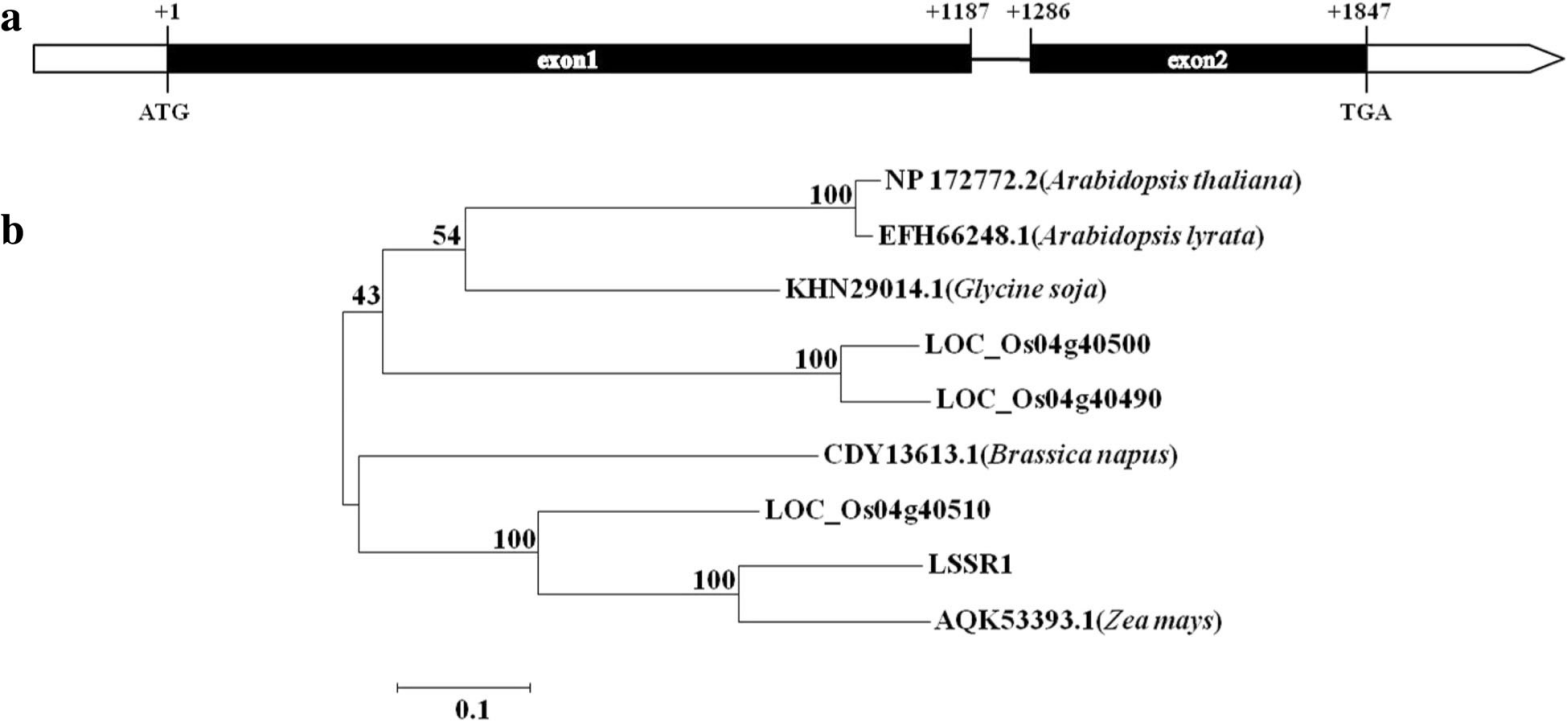
Bioinformatics analysis of LSSR1 (LOC_Os02g38260). **a** Diagram of the genomic locus of *LSSR1*. Exons are depicted as bars, the intron is depicted as line, and untranslated regions are indicated in white. **b** Phylogenetic analysis of LSSR1 and its homologs. The scale bar indicates the number of amino acid substitution per site

*LSSR1* encodes a putative GH5 cellulase of 582 amino acids. To identify the genomic structure of *LSSR1*, the genomic sequence and the full length CDS were compared. The result showed that the sequence consists in two exons and one intron (Fig. 2a), which is consistent with the information on the Rice Genome Annotation Project database (http://rice.plantbiology.msu.edu/).

**Fig. 3:**
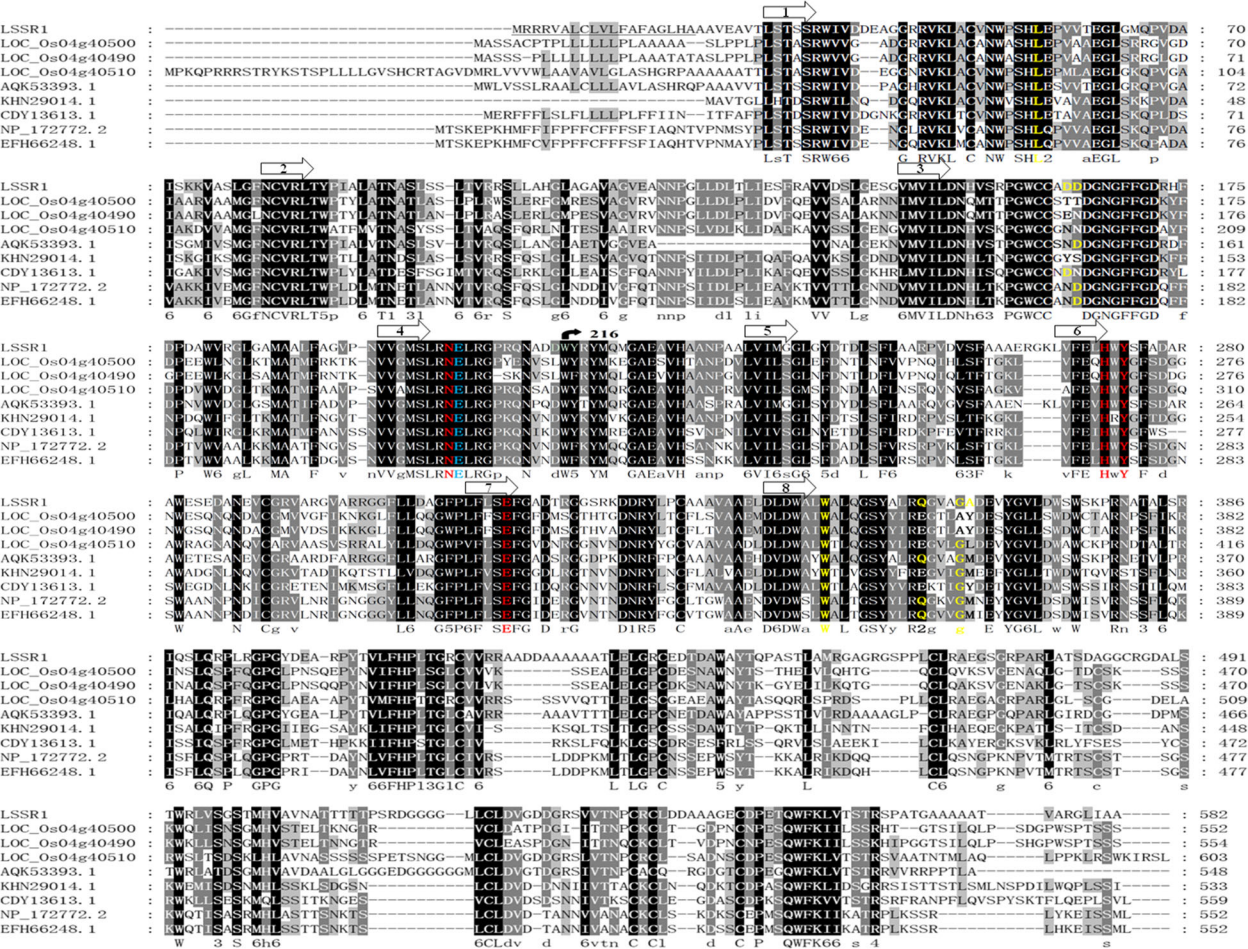
Sequence analysis of the cellusase LSSR1. The underlined 19 amino acids in the 5′ terminal are predicted as a signal peptide. The position of the β-strands of the (β/α)_8_ barrel are indicated by arrows above the alignment based on template c2zunB. Amino acids in yellow indicate the binding site, amino acids in red indicate the catalytic site, and the blue E (glutamic acid) may have both binding activity and catalytic activity. The blue E and the red E in β-strands 4 and 7 are the acid/base and nucleophile, respectively. The arrowed W^216^ (tryptophan) is the site where mutations in L1 lines begin

To examine the phylogenetic relationship between LSSR1 and its close homologs, BLASTP from the NCBI was used with the LSSR1 sequence as a query. With the threshold of identity (%) > 50% and non-presumptive proteins in the description, three paralogs of LSSR1 in rice (LOC_Os04g40490, LOC_Os04g40500 and LOC_Os04g40510) were found, whose encoding genes are adjacent on chromosome 4 (Fig. 2b). Coincidentally, these paralogs and LSSR1 are recorded as the four GH5 cellulases of rice in the Carbohydrate-Active enZYmes Database (CAZy) (http://www.cazy.org/GH5.html). Distinctly from *LSSR1*, the gene encoding the three paralogs is predicted to be expressed predominantly in rice seedling roots (*LOC_Os04g40490* and *LOC_Os04g40500*) or young leaves (*LOC_Os04g40510*) (Data from transcriptomic database Rice eFP Browser). Five orthologs of LSSR1 in other plants were found, including one in *Arabidopsis thaliana* (GenBank, NP 172772.2) and one in *Zea mays* (GenBank, AQK53393.1). The ortholog in *Zea mays* had the closest relationship with LSSR1 (Fig. 2b). The protein sequence of LSSR1 and its homologs are highly conserved. All of them share the two putative function-conserved glutamines (an acid/base and a nucleophile on β-strands 4 and 7, respectively) as GH5 hydrolases (Fig. 3). LSSR1 is predicted to have 12 active sites, including 4 catalytic sites, 7 binding sites and 1 residue having both activities, when considering the archaeon *Pyrococcus horikoshii* cellulase c2zunB as a template (Fig. 3). In addition, most of the predicted active sites are conserved in LSSR1 and its homologs (Fig. 3). Morever, LSSR1 contains a signal peptide at the N-terminal, which is predicted as a secretory protein (Fig. 3).

### LSSR1 is localized to the nucleus and cytoplasm through transient expression

**Fig. 4:**
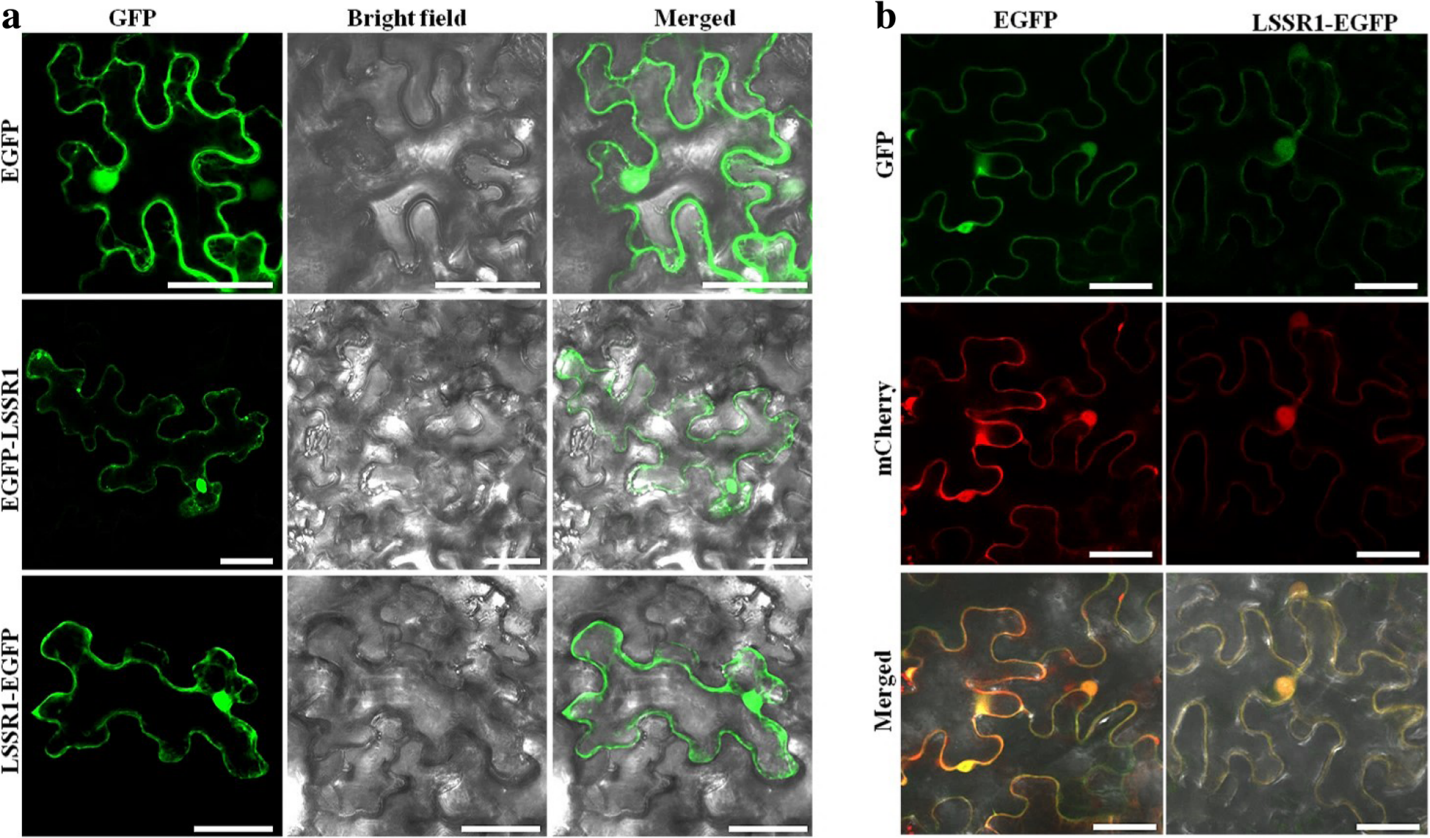
Subcellular localization of LSSR1. **a** Subcellular localization of EGFP-LSSR1 and LSSR1-EGFP. **b** Colocalization analysis between LSSR1 and mCherry. Bars = 50 μm

To evaluate whether LSSR1 is localized to the apoplast or other cell components, transient expression of the full-length CDS of *LSSR1* fused with *EGFP* in *N. benthamiana* leaves was conducted. Unexpectedly, the fluorescence signals of both LSSR1-EGFP and EGFP-LSSR1 proteins were observed in the nucleus and cytoplasm, which was the same localization of the EGFP protein alone control (Fig. 4a). To confirm this result, *LSSR1-EGFP* was co-expressed with *mCherry* alone in *N. benthamiana* leaves. As a result, LSSR1-EGFP could mainly be co-localized with mCherry (Fig. 4b). Since EGFP is pH sensitive, it may not fluoresce in the acidic environments such as the apoplast. Therfore, mCherry-LSSR1 and LSSR1-mCherry fused protein were transiently co-expressed with EGFP alone in the *N. benthamiana* leaves. The fluorescence of both mCherry-LSSR1 and LSSR1-mCherry could also be observed in the nucleus and cytosol, and could mainly be co-localized with the fluorescence of EGFP alone (Additional file 1: Figure S2). All these results suggest that LSSR1 can be localized to the nucleus and cytoplasm when heterologously over-expressed in *N. benthamiana*.

### *lssr1* exhibits significant low seed setting rate

**Fig. 5:**
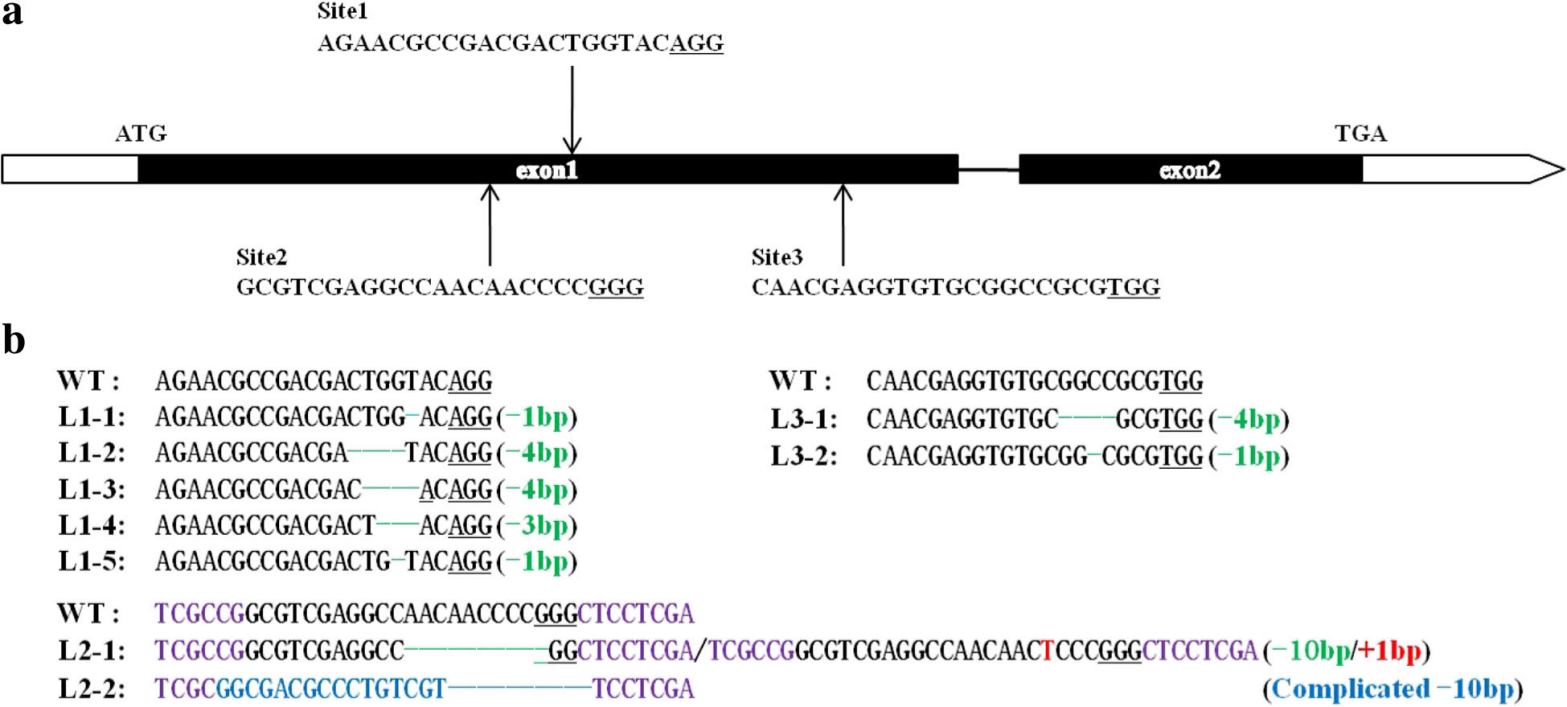
Targets and mutagenesis on rice *LSSR1* using CRISPR/Cas9 system. **a** Structure of *LSSR1* and the targets for knockout. **b** Effective targeted mutagenesis in the first exon. The underlined bases are PAMs, and the nucleotides in purple are flanking sequences of the targeted site

**Fig. 6:**
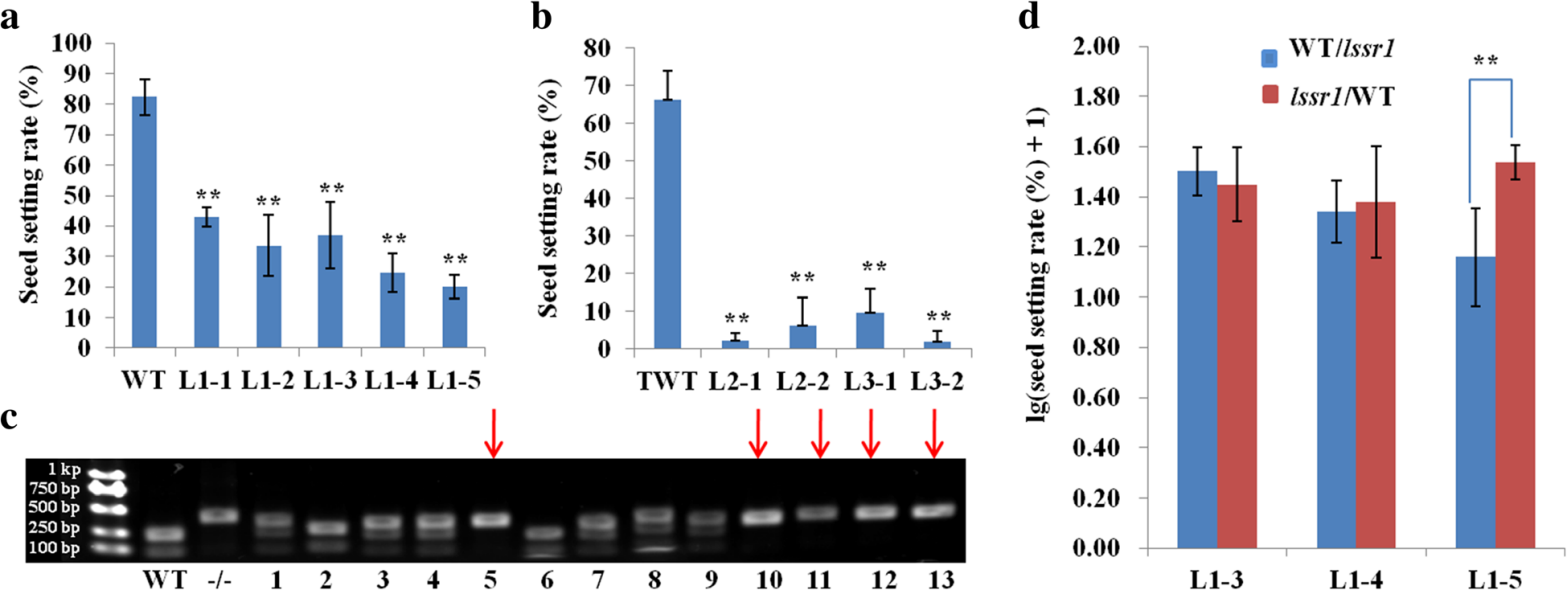
*lssr1* exhibits significant low seed setting rate. **a** Seed setting rate of *lssr1* lines with mutation in the first targeted site. Data are means ± SD from ≥ 6 individuals with 5 main panicles per plant. **b** Seed setting rate of the subsequent *lssr1* knockout individuals targeted in site2 and site3. TWT indicates the transgenic plant without mutation in *LSSR1*. Data are means + SD with 5 main panicles. **c** Partial image of the co-segregation analysis in segregated T_2_ populations. The genotypes were assessed by CAPS primer 38260 s1-F/R and restriction enzyme *Bse*NI. −/− is one of a T_2_ individual from homozygous *lssr1* line L1–5, and red arrows indicate T_2_ individuals with a low seed setting rate from heterozygous *lssr1*/*+* line L1–3 when compared with the WT. **d** Reciprocal crosses between the WT and *lssr1* mutant lines. Data are means ± SD of lg(seed setting rate(%) + 1) (for normalization) with ≥ 8 panicles. “**” indicates a significant difference at *P* < 0.01 by the Student’s t-test between the WT and L1 lines in **a**, between TWT and L2 or L3 individuals in **b**, and between the counterpart combinations in **d**

Since no study on the function of LSSR1 or its homologs has been done, a reverse genetic approach was used to examine the physiological role of *LSSR1*. The CRISPR/Cas9 system was used to create *lssr1* mutants. Initially, the first target in the middle of the first exon was selected to knock out *LSSR1* (Fig. 5a). 5 effective loss-of-function mutants were obtained, namely L1–1 (−1 bp/−3 bp), L1–2 (−4 bp/+), L1–3 (−4 bp/+), L1–4 (−3 bp/−), and L1–5 (−1 bp/−) (Fig. 5b). As the allelic −3 bp deletion in L1–1 was same as that of mutants L1–4, the −1 bp deletion homozygotes were chosen for seed-setting rate evaluation from the progeny of L1–1. L1–4 was predicted to produce LSSR1 peptides without the conserved W^216^ (tryptophan), while the remaining 4 mutants were assumed to generate truncated polypeptides as a result of non-triple-base deletion (Additional file 1: Figure S3). Significantly decreased seed-setting rates were observed in the homozygous progeny of *LSSR1* knockout lines when compared with that of the wild type (WT) (Fig. 6a, 7b). The seed setting rate of the WT was 82.43%, while only 42.94%, 33.56%, 37.02%, 24.68%, and 20.16% plump seeds were harvested in the homozygous T_3_ lines of L1–1~L1–5, respectively (Fig. 6a).

**Fig. 7:**
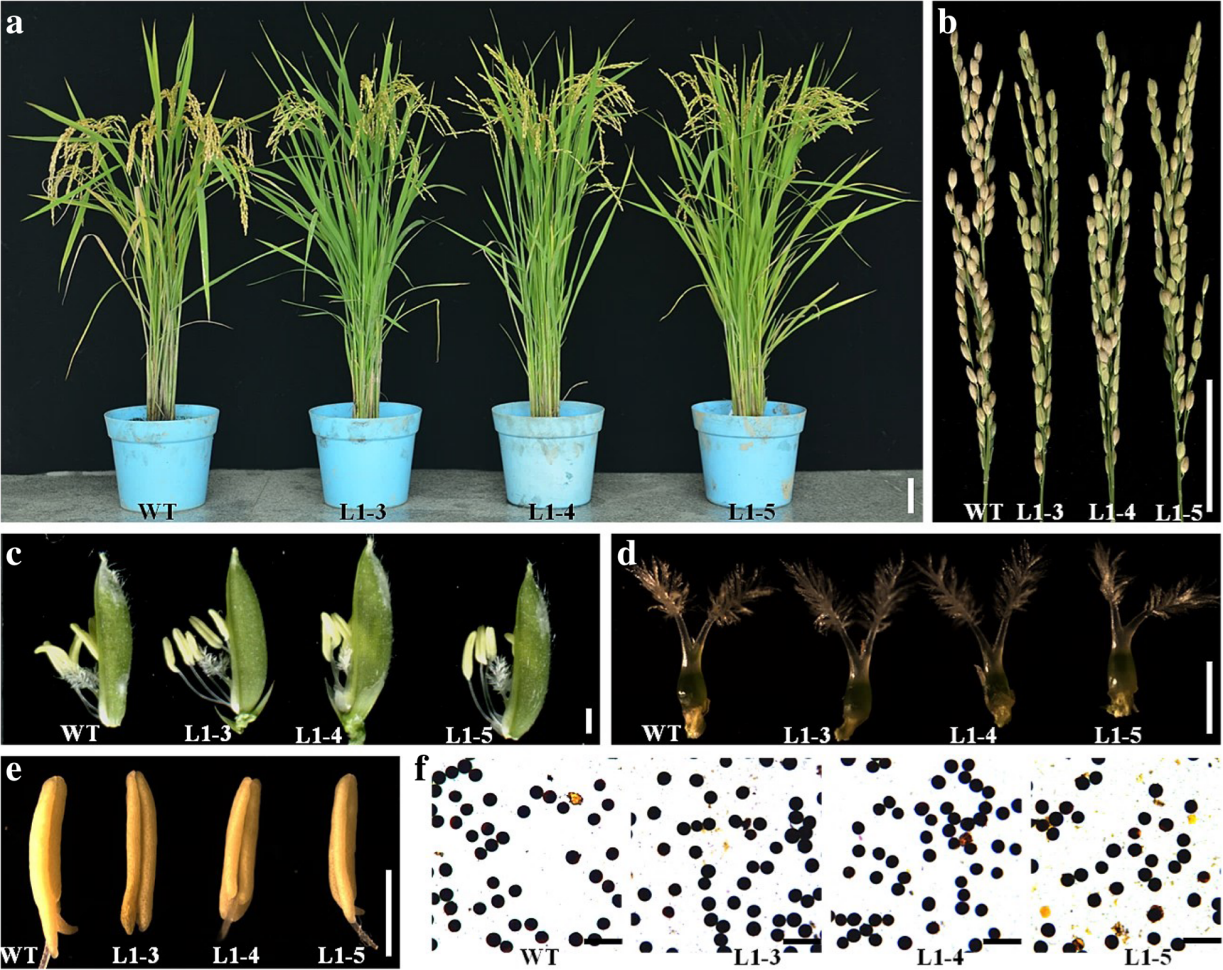
Phenotypic characterization of *lssr1* mutant lines. **a** Plant morphology of the WT and *lssr1* mutants at mature stage. **b** Mature panicles. **c** Florets. **d** Pistils. **e** Stamens. **f** I_2_-KI staining of pollen grains. Bars = 5 cm in **a** and **b**, 1 mm in **c-e**, and 100 μm in **f**

To confirm that the defective phenotype in the *lssr1* mutant lines resulted from the mutation in *LSSR1*, co-segregation analysis between the phenotype with the genotype in the segregated T_2_ population of L1–2 and L1–3 was conducted. Complete co-segregation was observed between the defected phenotype and the mutated genotype in L1–2 (*n* = 50) and L1–3 (n = 50) (Fig. 6c). Additionally, the plants in the segregated T_2_ population of L1–2 (χ^2^ = 1.24, *P* = 0.27) and L1–3 (χ^2^ = 0.26, *P* = 0.61) segregated plants of normal and low seed setting rate in a 3:1 ratio (Additional file 1: Table S2), suggesting that the low seed setting rate of *lssr1* lines is caused by a single recessive mutation in the sporophyte. To reconfirm the causality between the low seed setting rate with the mutated *LSSR1*, two additional CRISPR targets for *LSSR1* were selected to create new *lssr1* mutants (Fig. 5a). Four discernible frameshift mutants, namely L2–1 (−10 bp/+1 bp), L2–2 (Complicated −10 bp/−), L3–1 (−4 bp/−), and L3–2 (−1 bp/−) were obtained from site 2 and site 3, respectively (Fig. 5b). The seed setting rates of these mutants in T_0_ (1.96%~ 9.59%) were significantly lower than that of the transgenic plant without mutation in *LSSR1* (TWT, 66.25%) (Fig. 6b). These results indicate that the low seed-setting-rate phenotype of *lssr1* lines is exactly caused by the mutated *LSSR1*.

Reciprocal crosses between the WT and *lssr1* lines were conducted to determine whether it was the impaired stamens or pistils in the *lssr1* lines that resulted in a low seed setting rate. L1–3 (4 bp deletion), L1–4 (3 bp deletion), and L1–5 (1 bp deletion), the three lines with typical mutations, were chosen for reciprocal cross assay. When homozygous L1–5 plants were used as the pollen donors, the seed setting rate was significantly lower than that of the counterpart combination (Fig. 6d). This suggests that the male gamete may be responsible for reduced reproductive success in *lssr1* mutants. No difference in the other two pairs of combinations was found (Fig. 6d), which may be caused by excess pollination of the partially defective *lssr1* pollens.

### The number of pollen tubes that reached the *lssr1* ovules is significantly compromised

Because of the similar phenotype in all the independent and stable transgenic lines, L1–3, L1–4, and L1–5 homozygous T_3_ lines were used in subsequent experiments. *lssr1* lines showed normal vegetative and floret organs in their morphology (Fig. 7a, c). Also, there was no obvious difference in the morphology of the stamens and pistils between *lssr1* lines and the WT (Fig. 7d, e). Moreover, the pollen I_2_-KI staining appeared normal in the *lssr1* mutant lines when compared to that of the WT (Fig. 7f). Therefore, we supposed that the abortion in *lssr1* lines may occur in the process of fertilization, or embryo and endosperm development.

**Fig. 8:**
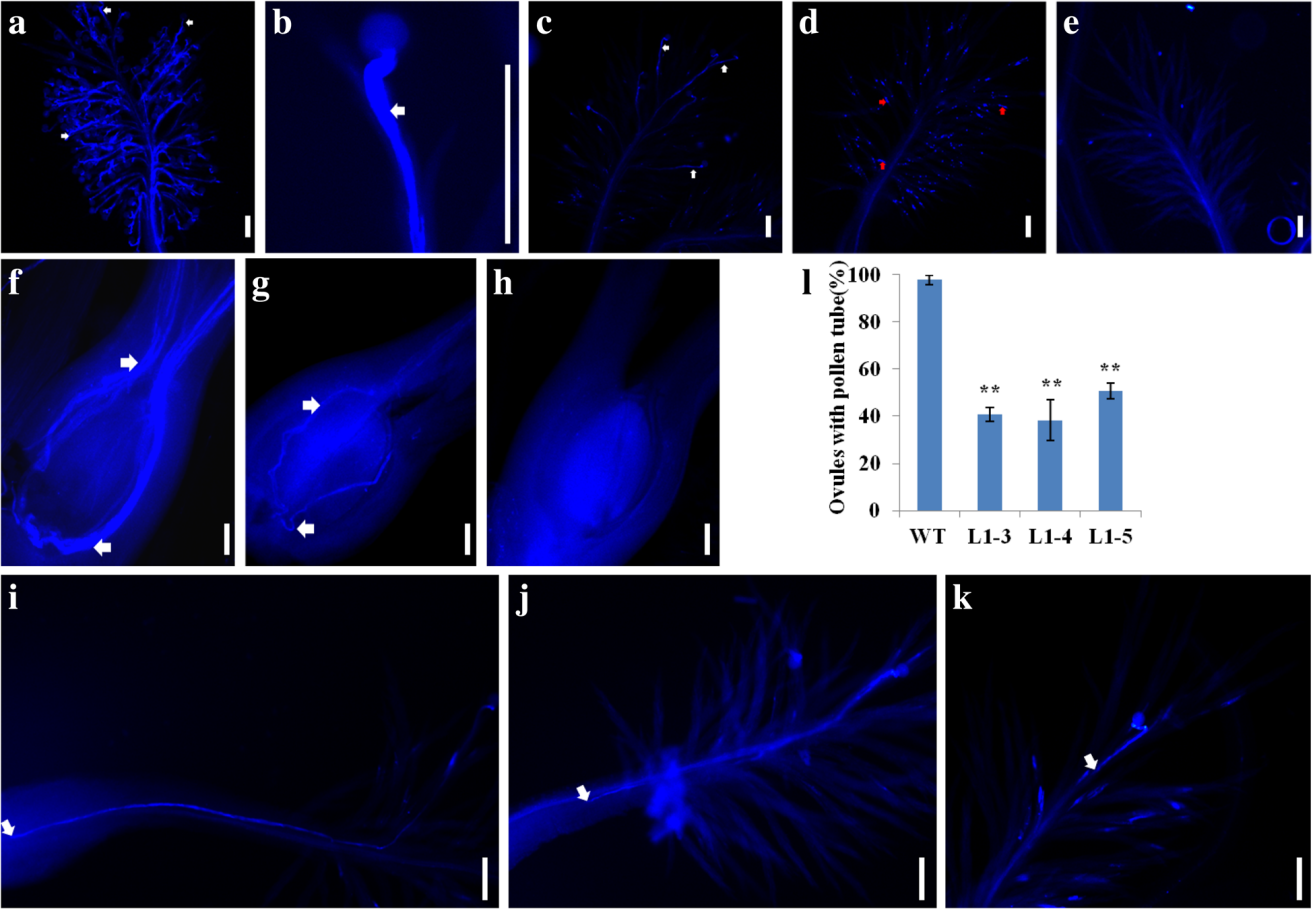
Pollen tube germination and growth of the WT and *lssr1* lines in vivo. **a** Aniline blue staining of the WT pistils at 5–30 min after flowering (MAF). **b** Amplified pollen tube of the WT. **c-e** Little or no germinated pollen grains were detected on the *lssr1* stigma at 5–30 MAF. **f** Multiple pollen tubes reached the ovules of the WT at 90–120 MAF. **g** and **h** few or zero pollen tubes reached the ovules of *lssr1* lines at 90–120 MAF. **i-k** Retardation of pollen tube growth in *lssr1* lines at 90–120 MAF. Bars = 100 μm. White arrows point to pollen tubes in **a-c**, **f** and **g**, and pollen tube tips in **i-k**. Red arrows point to pollen-tube-like dot lines on the stigma. **l** Quantification of ovaries with pollen tubes observed in the ovule at 90–120 MAF. Data are means ± SD from 3 replicates with ≥ 23 pistils per replicate, and “**” indicates a significant difference between the WT and *lssr1* lines at P < 0.01 by the Student’s t-test

To monitor the fertilization process in *lssr1* lines and the WT, the germination of pollen grains and pollen tube growth were compared in vivo. At 5–30 min after flowering (MAF), abundant pollen grains germinated on the WT stigmas, whereas few or zero germinated pollens were detected on most of the *lssr1* stigmas (Fig. 8a-e). On some of the *lssr1* stigmas, pollen-tube-like dot lines were observed, while on some of the *lssr1* stigmas, no signal could be found (Fig. 8d, e). At 90–120 MAF, about 97.85% of the WT pistils had pollen tube tips arriving at the basal ovules, but the number of *lssr1* ovules with pollen tubes was greatly reduced (L1–3, 40.77%; L1–4, 38.15%; L1–5, 50.79%) (Fig. 8l). In the ovules of the WT, abundant pollen tubes could be seen, while in *lssr1* ovules, few or zero pollen tubes were detected (Fig. 8f-h). Furthermore, some *lssr1* pistils had pollen tubes retarded in various positions (Fig. 8i-k). To evaluate whether the landing of pollen grains on the stigmas in *lssr1* lines is normal, pistils just after flowering in the room were collected and examined using the microscope (LEICA DM4 B, Germany). The number of pollen grains on all of the *lssr1* stigmas (Additional file 1: Figure S4) was more than 20, being sufficient to accomplish the fertilization (Sawada 1974). As the pollen exine plays a critical role in pollen adherence and hydration, scanning electron microscopy (SEM) observation was conducted to observe the morphology of pollen surface in the WT and *lssr1* lines. The morphologic surface of both the mature anther and pollen grains in *lssr1* lines showed no difference with that of the WT (Additional file 1: Figure S5). As such, the pollen grain adherence and hydration on the stigma may be normal in *lssr1* lines. These results suggest that the low seed-setting rate of the *lssr1* lines may result from abnormal pollen germination, pollen tube penetration, and pollen tube growth in the style.

### Conserved amino acid W^216^ impacts the function of LSSR1

**Fig. 9:**
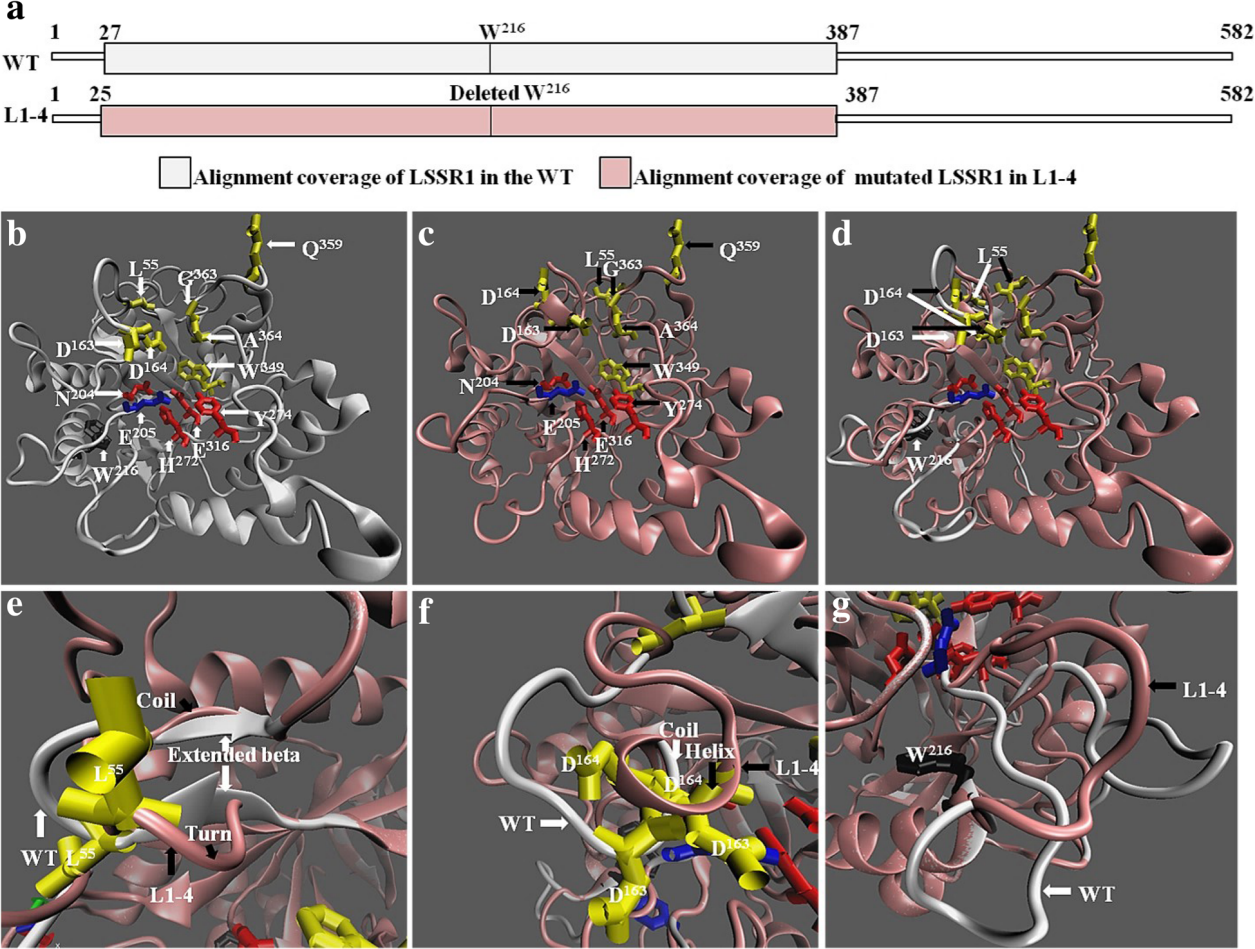
Predicted structure of LSSR1 in the WT and L1–4. The protein sequcence of LSSR1 in the WT and L1–4 were aligned with the same template c2zunB for structure prediction. **a** Alignment coverage of LSSR1 in the WT and L1–4. The three-dimensional (3D) model was constructed using these alignment covered sequences. **b** and **c** 3D model of the alignment covered sequence of LSSR1 in the WT and L1–4. **d** The merged structure in **b** and **c**. **e-g** Amplified image of the differential parts between the normal LSSR1 in the WT and the mutated LSSR1 in L1–4. White arrows point to residues (**b**, **d**) or domains (**e-g**) of the WT LSSR1, and black arrows point to residues (**c**, **d**) or domains (**e-g**) of the mutated LSSR1 in L1–4. Residues in yellow indicate the binding site, and in red indicate the catalytic site. The blue E^205^ (glutamine) may have both binding activity and catalytic activity, and the black W^216^ (tryptophan) is deleted in L1–4. E^205^ and E^316^ are the acid/base and the nucleophile, respectively

Sequence analysis indicates that W^216^ (tryptophan) is a conserved amino acid of LSSR1 and its homologs (Fig. 3). The deletion of W^216^ (tryptophan) on LSSR1 also results in a low seed setting rate in L1–4 (Additional file 1: Figure S3; Fig. 6a, 7b), implying that W^216^ is a functionally important residue for LSSR1. To explore that if the proper folding of LSSR1 is affected by this residue, the three-dimensional (3D) structure of the functional dormain of both the WT LSSR1 and L1–4 LSSR1 were predicted using Phyre2 with the same template (Fig. 9a). Althrough the overall structure and catalytic groove on the W^216^-deleted LSSR1 seem to be similar to that of the WT LSSR1, some parts of the binding surface of the mutated LSSR1 change (Fig. 9b-d). In W^216^-deleted LSSR1, the extended betas on both sides of L^55^ (leucine) in the WT LSSR1 changes to be a coil and a turn, the coiled D^163/164^ (aspartatic acid) along with a adjacent residue on both sides becomes a helix, and the loop structure between the acid/base E^205^ (glutamic acid) and the deleted W^216^ looses (Fig. 9e-g). These results suggest that W^216^ may have a great effect on the spatial structure and proper function of LSSR1.

## Discussion

In this study, we describe a novel cellulase encoding gene *LSSR1* in rice. Dysfunction of *LSSR1* results in a low seed setting rate, but the morphology of the vegetative and reproductive organs, as well as the pollen I_2_-KI staining, appears normal in *lssr1* mutant lines (Fig. 6a, b; 7). Our cytological studies suggest that it is most likely the blockage of fertilization that leads to the decreased seed setting rate in *lssr1* lines. The majority of *lssr1* pistils had few or no germinated pollen grains adhering to the stigma at 5–30 MAF, but pollen-tube-like dot lines remained on some *lssr1* stigmas (Fig. 8a-e). At 90–120 MAF, the number of *lssr1* ovules with pollen tubes was significantly lower than that of the WT (Fig. 8l). Additionally, retarded pollen tubes were detected in *lssr1* pistils at 90–120 MAF (Fig. 8i-k). As more than 20 pollen grains could be observed on the *lssr1* stigmas just after flowering (Additional file 1: Figure S4), we deduce that the landing of pollens on the stigma in *lssr1* lines does not account for their abnormal fertilization. In Arabidopsis (*Arabidopsis thaliana*), the lipophilic molecules in the pollen exine mediate the pollen-stigma adhesion (Zinkl et al. 1999), while the aperture number influences pollen survival against water inflow (Prieu et al. 2016), suggesting that the pollen exine is of great importance for pollen adherence and hydration on the stigma. Nonetheless, the morphology of the pollen epidermal surface of *lssr1* lines showed no difference with that of the WT (Additional file 1: Figure S5h1-h4). Therefore, the pollen adherence and hydration on the stigma in *lssr1* lines are probably normal. In conclusion, the arrested fertilization in *lssr1* lines may result from the aborted pollen grain germination, failed pollen tube penetration, and unsuccessful elongation of pollen tubes to the ovules. Additional experiments are needed to validate these results and find out the critical barrier for the successful fertilization in *lssr1* lines.

Any deviation during fertilization in flowering plants could result in sterility. The OsMLO12 protein contains seven transmembrane motifs, which regulates pollen hydration through an interaction with calmodulin in the cytosol (Yi et al. 2014). *Osmlo12* pollens matured normally with three nuclei, but could not germinate in vitro or in vivo due to a failure in hydration (Yi et al. 2014). The disruption of rice aspartic protease OsAP65 caused the dysfunction in pollen tube germination and elongation (Huang et al. 2013). AtOFT1, a putative protein O-fucosyltransferase in *Arabidopsis*, promotes pollen tube penetration through the stigma-style interface (Smith et al. 2018). *oft1* pollen tubes could germinate normally, but their ability to penetrate through the stigma during fertilization was largely compromised (Smith et al. 2018). BUPS1/2, as the receptors of signals from the female cells in *Arabidopsis*, coordinate the pollen tube integrity and sperm release by interacting with RAF4/19 and ANXUR1/2 (Ge et al. 2017). *bups1 bups2* pollen tubes ruptured immediately upon germination (Ge et al. 2017). All of these genes regulate plant fertility through gametophyte and showed segregation distortion in the progeny of heterozygous mutants. However, the segregation ratios in the progeny of *lssr1* heterozygous mutants were in accordance with Mendelian inheritance (Additional file 1: Table S2), indicating that *LSSR1* control rice fertility through the sporophyte.

Cellulose, the main load-bearing polysaccharide in all cell walls of higher plants (Mélida et al. 2009), may be the critical barrier for rice fertilization. Previous research has suggested that the pollen tube must penetrate the cuticle and cell wall on the stigma to create an entry for further penetration (Jiang 2005). In this process, wall hydrolases, including cellulases, either derived from the tapetum cells of developing anthers or rapidly synthesized by pollens, play a significant role. ZmXYN1 is an endoxylanase in maize, whose mRNA is located in the tapetum cells enclosing the developing pollen in anthers, while the mature 35-kDa endoxylanase is the predominant protein in the maize pollen coat (Wu et al. 2002). Xyl-less (Transgenic lines containing little or no xylanase in the pollen coat) pollen tubes germinated and elongated comparably with that of the WT pollen tubes, but did not penetrate into the silk as efficiently as pollen tubes of the WT (Suen and Huang 2006). Another major protein in the maize pollen coat is *Zm*GLA3, a 70-kDa β-glucanase, which is also derived from the tapetum in developing anthers and may hydrolyze the stigma wall for pollen tube entry (Suen et al. 2003). LSSR1 is predicted as a cellulase. The qRT-PCR analysis showed that *LSSR1* is predominantly expressed in anthers during the developmental stage of microsporogenesis, including the premeiosis, meiosis, and single-cell pollen stages (Fig. 1b-f). Like EGFP or mCherry alone control, the encoded protein of *LSSR1* can be localized to the nucleus and cytoplasm in *N. benthamiana* (Fig. 4; Additional file 1: Figure S2), which is inconsistent with the putative secretion of LSSR1. Therefore, *mCherry*-fused complementation lines will be created to confirm the localization of LSSR1 in our future study. We suppose that LSSR1 is synthesized in the tapetum cells and transported to the developing microspores, and functions during rice fertilization. However, we have not yet obtained the purified functional protein to evaluate its catalytic activity and substrates.

When comparing with template c2zunB, 5 catalytic sites and 8 binding sites of LSSR1, including the acid/base and the nucleophile, were predicted, respectively (Fig. 3). In addition, some other amino acid residues conserved in LSSR1 and its homologs were marked with black background (Fig. 3). A deletion of the conserved W^216^ (tryptophan) in L1–4 leads to the defective phenotype, just as other garbled and truncated mutations do (Additional file 1: Figure S3; Fig. 6a, 7b). This implies that W^216^ is a functionally important residue for LSSR1. Structure analysis showed some difference on the binding surface between the WT LSSR1 and W^216^-deleted LSSR1 (Fig. 9d-g). These results indicate that the proper folding of LSSR1 and its normal function is largely impacted by the conserved W^216^. Furthermore, elucidating the function of the putative active sites in LSSR1 will also be the subject of future studies.

Morever, the partial defect of seed setting in *lssr1* lines indicates that other cellulases or hydrolases with cellulase activity in or on the surface of pollen grains may function during rice fertilization. There are 25 rice GH5 proteins in the CAZy database, most of which are putative mannosidases. The *β*-glucosidase GH5BG is one of the GH5 proteins in rice, which may be a regulator in response to stress in rice seedlings (Opassiri et al. 2007). No functional analysis has been made in other rice GH5 proteins. LSSR1 and its three paralogs (LOC_Os04g40490, LOC_Os04g40500 and LOC_Os04g40510) are predicted to be cellulases (Fig. 2b), but they may have various expression patterns according to the data from the Rice eFP Browser. More studies are required to clarify whether the paralogs of LRR1 or other hydrolases coordinate with LRR1 to hydrolyze celluloses in the papillar cell walls.

Overall, this study provides strong evidence to suggest that the novel gene *LSSR1* plays a critical role in controlling the seed setting rate of rice. It most likely does this by controlling the fertilization process. No cellulase gene had ever been linked to rice seed setting rate previously. The discovery of a low seed setting rate in *lssr1* mutants provides a unique opportunity to use cell biological techniques (such as in vitro pollen tube growth assay and semi-in-vivo assay) and robust genetics to investigate how GH5 cellulases function during plant fertilization, as well as in rice seed setting.

## Additional file

Additional file 1: **Figure S1.** The information of *LSSR1* (*LOC_Os02g38260*) from transcriptomic database Rice eFP Browser (http://www.bar.utoronto.ca/efprice/cgi-bin/efpWeb.cgi). Different stages of panicle and seed development have been categorized according to panicle length and days after pollination (dap), respectively, based on landmark developmental events as follows: 0–3 cm, floral transition and floral organ development (P1); 3–10 cm, meiotic stage (P2 and P3); 10–15 cm, young microspore stage (P4); 15–22 cm, vacuolated pollen stage (P5); 22–30 cm, mature pollen stage (P6); 0–2 dap, early globular embryo (S1); 3–4 dap, middle and late globular embryo (S2); 5–10 dap, embryo morphogenesis (S3); 11–20 dap, embryo maturation (S4); 21–29 dap, dormancy and desiccation tolerance (S5). These stage specifications are approximations based on information from Itoh et al., 2005, Plant Cell Physiol, 46, 23–47. **Figure S2.** Co-localization analysis between LSSR1 and EGFP. Bars = 50 μm. **Figure S3.** LSSR1 protein sequences in WT and *lssr1* lines. Amino acids and dotted lines marked with yellow background represent mutation site or domains. L2–1-1 and L2–1-2 indicate the two allelic mutations in L2–1. **Figure S4.** Pollens on the stigma of WT and *lssr1* lines. 30 pistils just after flowering in the room were collected for photographing in every line. Bar, 500 μm. **Figure S5.** Scanning electron microscopy (SEM) analysis of the surfaces of anthers and pollen grains in the WT and *lssr1* lines. **a1-a4**, Integrated anthers. **b1-b4**, Crushed anthers. **c1-c4**, Anther epidermal surfaces. **d1-d4**, Enlarged anther epidermal surfaces. **e1-e4**, Anther inner surfaces. **f1-f4**, Enlarged anther inner surfaces. **g1-g4**, Pollen grains. **h1-h4**, Enlarged pollen epidermal surfaces. Bars: **a1-a4** and **b1-b4**, 200 μm; **c1-c4**, **d1-d4**, **e1-e4**, **f1-f4**, and **h1-h4**, 2 μm; **g1-g4**, 5 μm. **Table S1** Primers used in this study. **Table S2** Segregation analysis of heterozygous *lssr1*/*+* lines in T_2_. (DOCX 4700 kb)

## Abbreviations

3D: Three-dimensional
Ca^2+^_cyt_: Cytoplasmic calcium concentration
CAPS: Cleaved polymorphic amplified sequence
ECM: Extracellular matrix
FAA: Formalin-aceto-alcohol
GH: Glycosyl hydrolase
MAF: Minutes after flowering
SEM: Scanning electron microscopy
STT: Style transmission tissue
TT: Transmitting tract
