# Supplementary material for: LSSR1 facilitates seed setting rate by promoting fertilization in rice
Source: Rice (N Y). 2019 May 9;12:31. doi: 10.1186/s12284-019-0280-3 (PMC6509318; doi:10.1186/s12284-019-0280-3)
Supplement: Supplementary file 1 — Figure S1. The information of LSSR1 (LOC_Os02g38260) from transcriptomic database Rice eFP Browser (http://www.bar.utoronto.ca/efprice/cgi-bin/efpWeb.cgi). Different stages of panicle and seed development have been categorized according to panicle length and days after pollination (dap), respectively, based on landmark developmental events as follows: 0–3 cm, floral transition and floral organ development (P1); 3–10 cm, meiotic stage (P2 and P3); 10–15 cm, young microspore stage (P4); 15–22 cm, vacuolated pollen stage (P5); 22–30 cm, mature pollen stage (P6); 0–2 dap, early globular embryo (S1); 3–4 dap, middle and late globular embryo (S2); 5–10 dap, embryo morphogenesis (S3); 11–20 dap, embryo maturation (S4); 21–29 dap, dormancy and desiccation tolerance (S5). These stage specifications are approximations based on information from Itoh et al., 2005, Plant Cell Physiol, 46, 23–47. Figure S2. Co-localization analysis between LSSR1 and EGFP. Bars = 50 μm. Figure S3. LSSR1 protein sequences in WT and lssr1 lines. Amino acids and dotted lines marked with yellow background represent mutation site or domains. L2–1-1 and L2–1-2 indicate the two allelic mutations in L2–1. Figure S4. Pollens on the stigma of WT and lssr1 lines. 30 pistils just after flowering in the room were collected for photographing in every line. Bar, 500 μm. Figure S5. Scanning electron microscopy (SEM) analysis of the surfaces of anthers and pollen grains in the WT and lssr1 lines. a1-a4, Integrated anthers. b1-b4, Crushed anthers. c1-c4, Anther epidermal surfaces. d1-d4, Enlarged anther epidermal surfaces. e1-e4, Anther inner surfaces. f1-f4, Enlarged anther inner surfaces. g1-g4, Pollen grains. h1-h4, Enlarged pollen epidermal surfaces. Bars: a1-a4 and b1-b4, 200 μm; c1-c4, d1-d4, e1-e4, f1-f4, and h1-h4, 2 μm; g1-g4, 5 μm. Table S1 Primers used in this study. Table S2 Segregation analysis of heterozygous lssr1/+ lines in T2. (DOCX 4700 kb) [file 12284_2019_280_MOESM1_ESM.docx]

**Additional file**

Rice

***LSSR1* facilitates seed setting rate by promoting fertilization in rice**

Xiaojiao Xiang^1, 2^, Peipei Zhang^1^, Ping Yu^1^, Yingxin Zhang^1^, Zhengfu Yang^1, 2^, Lianping Sun^1^, Weixun Wu^1^, Riaz Muhammad Khan^1^, Adil Abbas^1^, Shihua Cheng^1,^ *, Liyong Cao^1,^ *

^1^Key Laboratory for Zhejiang Super Rice Research and State Key Laboratory of Rice Biology, China National Rice Research Institute, Hangzhou, 310006, China

^2^National Key Laboratory of Crop Genetic Improvement, Huazhong Agricultural University, Wuhan, 430070, China

* Corresponding authors: Liyong Cao, 86-571-6337-0329, [caoliyong1966@163.com](mailto:caoliyong1966@163.com); Shihua Cheng, 86-571-6337-0188, chengshihua@caas.cn

**Additional figures**


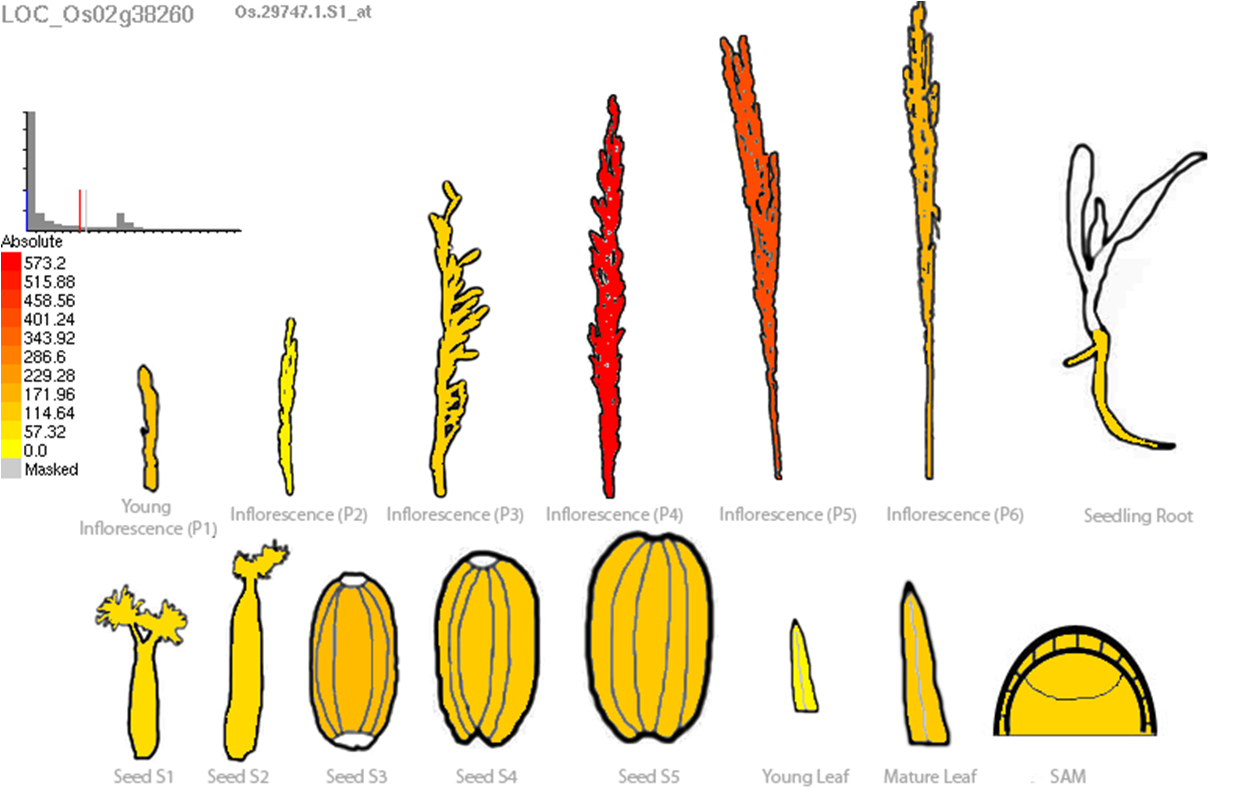


**Figure S1** The information of *LSSR1* (*LOC_Os02g38260*) from transcriptomic database Rice eFP Browser (http://www.bar.utoronto.ca/efprice/cgi-bin/efpWeb.cgi). Different stages of panicle and seed development have been categorized according to panicle length and days after pollination (dap), respectively, based on landmark developmental events as follows: 0-3 cm, floral transition and floral organ development (P1); 3-10 cm, meiotic stage (P2 and P3); 10-15 cm, young microspore stage (P4); 15-22 cm, vacuolated pollen stage (P5); 22-30 cm, mature pollen stage (P6); 0-2 dap, early globular embryo (S1); 3-4 dap, middle and late globular embryo (S2); 5-10 dap, embryo morphogenesis (S3); 11-20 dap, embryo maturation (S4); 21-29 dap, dormancy and desiccation tolerance (S5). These stage specifications are approximations based on information from Itoh et al., 2005, Plant Cell Physiol, 46, 23-47

**
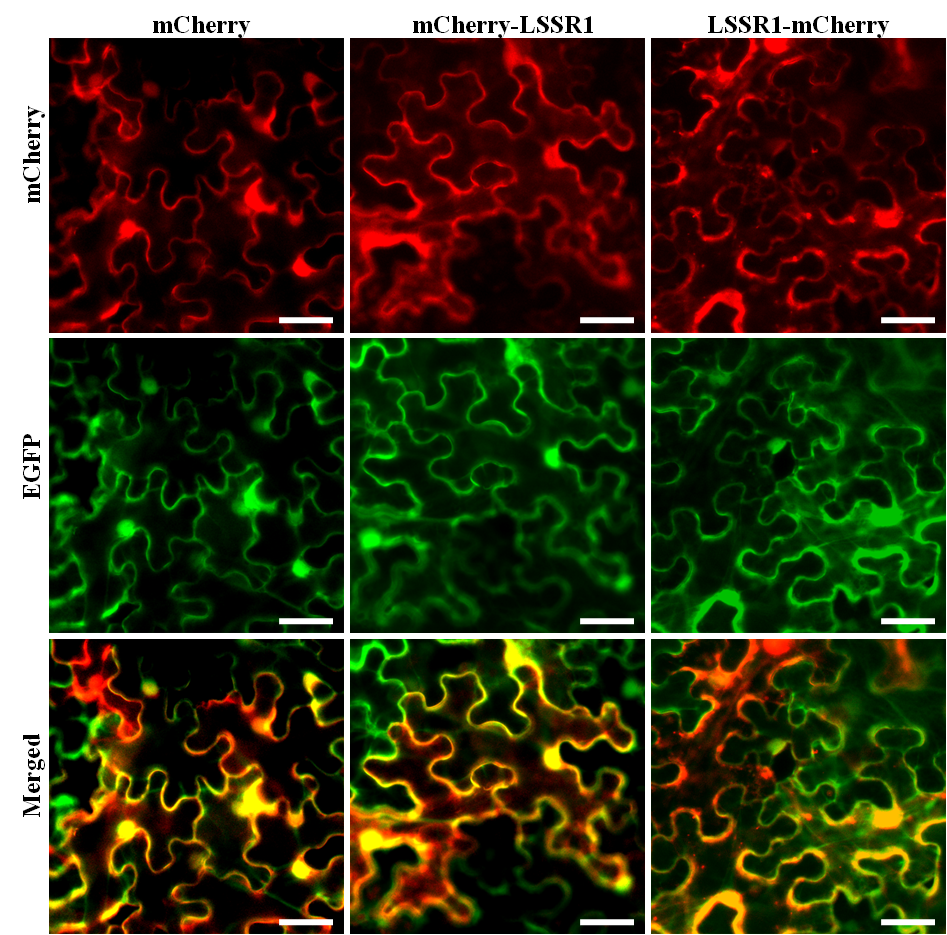
**

**Figure S2** Co-localization analysis between LSSR1 and EGFP. Bars = 50 μm


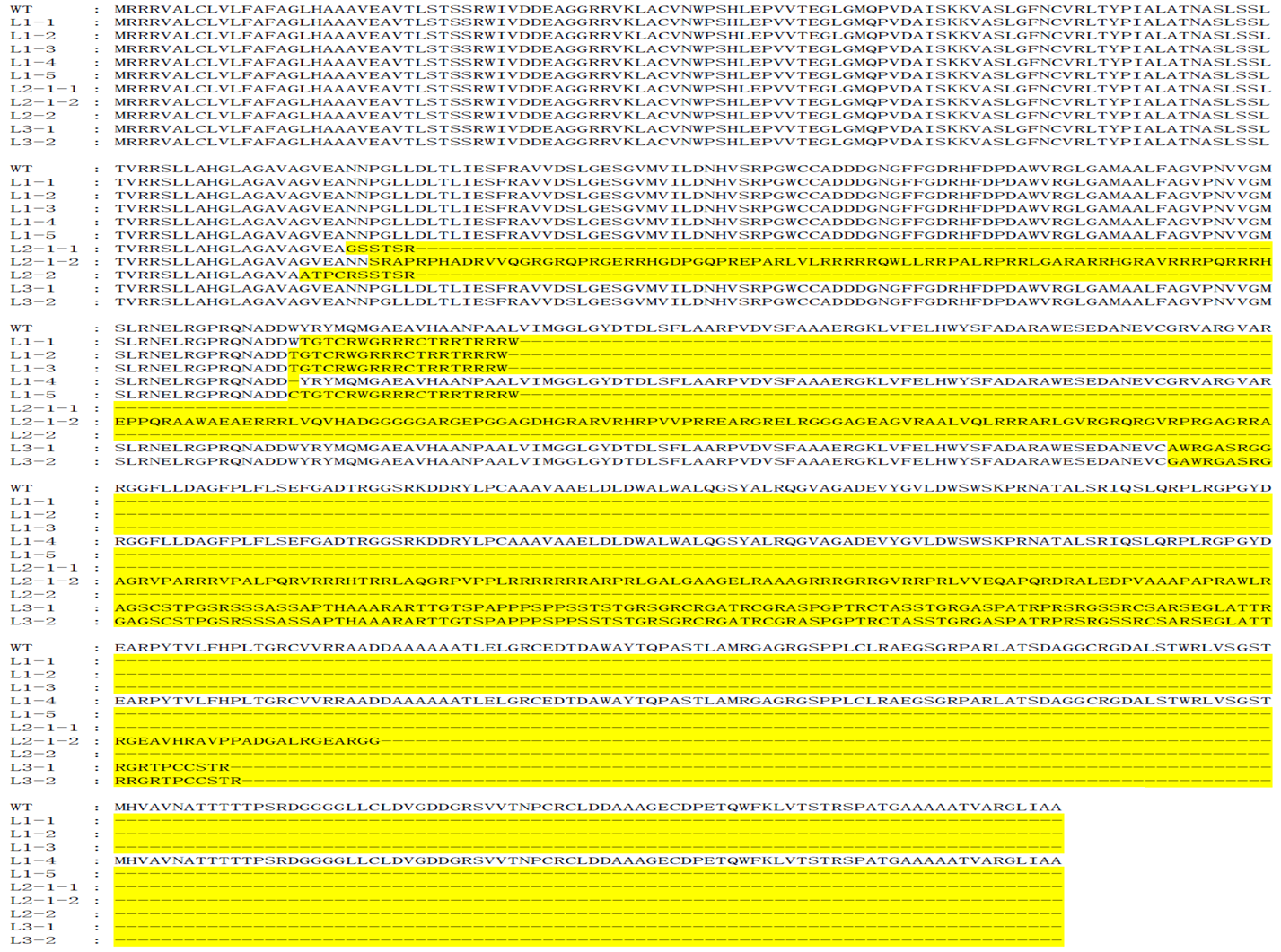


**Figure S3** LSSR1 protein sequences in WT and lssr1 lines. Amino acids and dotted lines marked with yellow background represent mutation site or domains. L2-1-1 and L2-1-2 indicate the two allelic mutations in L2-1


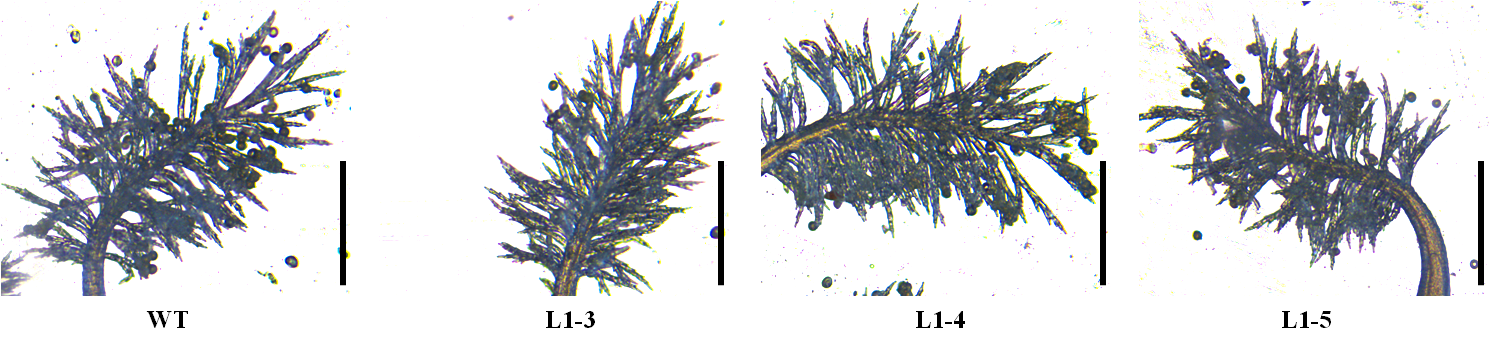


**Figure S4** Pollens on the stigma of WT and *lssr1* lines. 30 pistils just after flowering in the room were collected for photographing in every line. Bar, 500μm


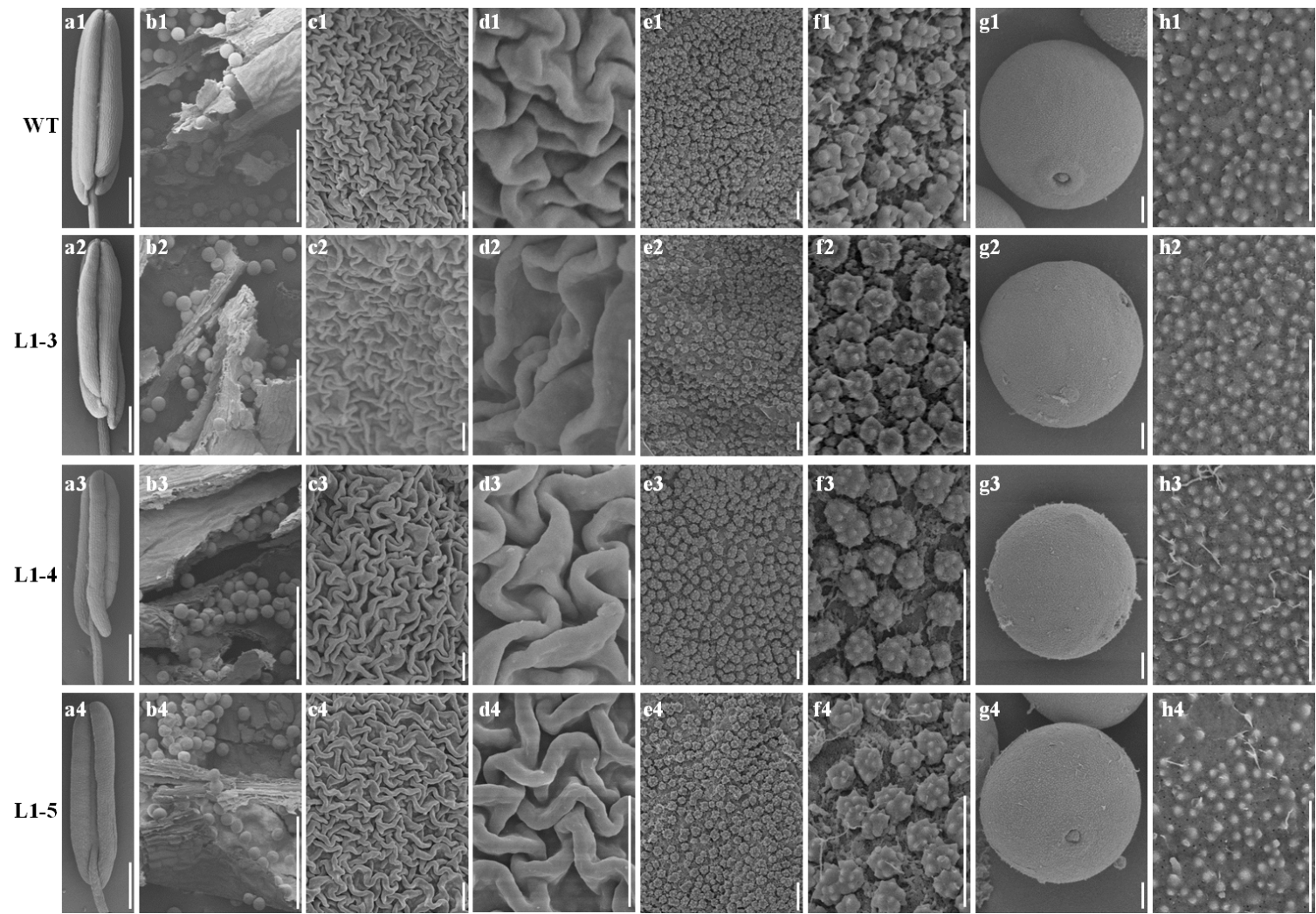


**Figure S5** Scanning electron microscopy (SEM) analysis of the surfaces of anthers and pollen grains in the WT and *lssr1* lines. **a1-a4**, Integrated anthers. **b1-b4**, Crushed anthers. **c1-c4**, Anther epidermal surfaces. **d1-d4**, Enlarged anther epidermal surfaces. **e1-e4**, Anther inner surfaces. **f1-f4**, Enlarged anther inner surfaces. **g1-g4**, Pollen grains. **h1-h4**, Enlarged pollen epidermal surfaces. Bars: **a1-a4** and **b1-b4**, 200 μm; **c1-c4**, **d1-d4**, **e1-e4**, **f1-f4**, and **h1-h4**, 2 μm; **g1-g4**, 5 μm

**Additional tables**

**Table S1** Primers used in this study

| **Primer name** | **Sequence ( 5'-3' )** | **Purpose** | **Comment** |
| --- | --- | --- | --- |
| ActinqPCR-F | TGCTATGTACGTCGCCATCCAG | qRT-PCR | Cited from Zhang et al (2017) |
| ActinqPCR-R | AATGAGTAACCACGCTCCGTCA |  |  |
| 38260qPCR-F | GTGCATTCGTCATCAGAGAATC |  | Searched from the qPCR Primer Database (https://biodb.swu.edu.cn/qprimerdb/) |
| 38260qPCR-R | GACACGAACTCTCTAACTTCCA |  |  |
| 38260GFP-F | CGCTCGCCTCACTGCATTTC | Subcellular localization |  |
| 38260GFP-R | CGCAGCAATTAGCCCTCGCG |  |  |
| 38260s1-F | CGACCTCACGCTGATCGAGTC | Sequencing, genotyping | Target site1; For genotyping, the PCR products were digested by the restriction enzyme *Bse*NI |
| 38260s1-R | CGAGGAACGACAGGTCGGTG |  |  |
| hygromycin-F | GTATCACTGGCAAACTGTGATG | T-DNA detection |  |
| hygromycin-R | GAGTACTTCTACACAGCCATCG |  |  |
| Cas9i2-F | CTCAAAAGCGTCAAGGAACTGC |  | Provided by Wuhan BioRun Technology Co. Ltd |
| Cas9i2-R | TAATGTTTTCTGCCTGCTCCCT |  |  |
| 38260s2-F | CTGCATTTCTATCCCGGCCAT | Sequencing | Target site2 and site3, respectively |
| 38260s2-R | GTTGTCCAGGATCACCATGACG |  |  |
| 38260s3-F | TGGTACAGGTACATGCAGATGG |  |  |
| 38260s3-R | CCGAACTCGCTGAGGAAGAG |  |  |

**Table S2** Segregation analysis of heterozygous *lssr1*/*+* lines in T_2_

| **Mutant lines** | **Normal individuals** | **Low fertile individuals** | **Expected ratio** | **χ2** | ***P*** |
| --- | --- | --- | --- | --- | --- |
| L1-2 | 252 | 96 | 3:1 | 1.24 | 0.27 |
| L1-3 | 135 | 49 | 3:1 | 0.26 | 0.61 |
